# What Klein’s “Semantic Gradient” Does and Does Not Really Show: Decomposing Stroop Interference into Task and Informational Conflict Components

**DOI:** 10.3389/fpsyg.2016.00249

**Published:** 2016-02-26

**Authors:** Yulia Levin, Joseph Tzelgov

**Affiliations:** ^1^Automaticity Skill and Consciousness Lab, Department of Psychology, Ben-Gurion University of the NegevBeer Sheva, Israel; ^2^Department of Brain and Cognitive Sciences, Zlotowski Center for Neuroscience, Ben-Gurion University of the NegevBeer Sheva, Israel; ^3^Achva Academic CollegeArugot, Israel

**Keywords:** Stroop task, automaticity, semantic gradient, task conflict, informational conflict

## Abstract

The present study suggests that the idea that Stroop interference originates from multiple components may gain theoretically from integrating two independent frameworks. The first framework is represented by the well-known notion of “semantic gradient” of interference and the second one is the distinction between two types of conflict – the task and the informational conflict – giving rise to the interference (MacLeod and MacDonald, 2000; Goldfarb and Henik, 2007). The proposed integration led to the conclusion that two (i.e., orthographic and lexical components) of the four theoretically distinct components represent task conflict, and the other two (i.e., indirect and direct informational conflict components) represent informational conflict. The four components were independently estimated in a series of experiments. The results confirmed the contribution of task conflict (estimated by a robust orthographic component) and of informational conflict (estimated by a strong direct informational conflict component) to Stroop interference. However, the performed critical review of the relevant literature (see General Discussion), as well as the results of the experiments reported, showed that the other two components expressing each type of conflict (i.e., the lexical component of task conflict and the indirect informational conflict) were small and unstable. The present analysis refines our knowledge of the origins of Stroop interference by providing evidence that each type of conflict has its major and minor contributions. The implications for cognitive control of an automatic reading process are also discussed.

## Introduction

A landmark cognitive task in the field of automaticity research is rightfully considered the Stroop task (Stroop, 1935). In the classic variation, participants are required to name the color of the ink in which a word stimulus is presented. It usually takes more time for participants to name the color when it is incompatible with the meaning of the word (i.e., when the stimulus is incongruent, e.g., the word BLUE written in red ink) than when the meaning of the word is color-unrelated (e.g., the word DOG) or when the stimulus is meaningless (e.g., a letter string such as XXXX). This finding is known as the *interference effect* and it is commonly believed to occur because there is an incompatibility between the meaning of the word and a color of the ink the word is presented in. However, as will be further explained in more detail, the observed incompatibility is only a visible part—the “top of an iceberg”—which should not be confused with a primary origin of the Stroop interference effect. To preview the following discussion, it is our belief that Stroop interference should generally be viewed as a behavioral expression of the fact that stimulus words are being automatically read. Let us briefly discuss the two main points of this proposal—the key role of reading and the automaticity of reading—in turn. The key role of reading in the Stroop task is emphasized by the fact that only by means of reading can the meaning be extracted from a visual lexical symbol (i.e., a word). That is, even if accepting a somewhat limited view of the Stroop interference as representing an incompatibility effect (see further discussion below), it is clear that such incompatibility can only arise if the word stimulus has been read. However, why should the word stimuli be read at all if the required task is to name the color of the ink? Based on the revised definition of automaticity proposed by Tzelgov (1997) and Perlman and Tzelgov (2006), according to which a process is automatic if it occurs in spite of the fact that it is not required for the successful performance of the task, the words are read automatically. Note, that in contrast to early views of automaticity (Posner and Snyder, 1975; Shiffrin and Schneider, 1977; Hasher and Zacks, 1979), the definition proposed by Tzelgov, and Tzelgov and Perlman does not involve relying on other cognitive constructs such as attention or awareness. Instead, it emphasizes the ballistic feature of automatic processes—their inclination to run to full completion once they have been trigged by the stimulus they are highly associated with (Bargh, 1989). Hence, the Stroop situation is unique in that it provides tangible evidence of automaticity of the reading process that can be measured and explored.

Although it has been extensively investigated for almost 80 years, the behavior observed during performance of the Stroop task has yet to be fully understood. Findings of different studies, however, have led to an important recognition that the interference effect has multiple components. Currently, there are at least two different theoretical frameworks indicating multiple origins of the interference effect. Although these frameworks seem at first glance to be conceptually different, we believe their integration, as carried out in the experiments herein, is crucial and is a very important step toward our understanding of automaticity of reading in general, and of the Stroop phenomenon in particular. The first of these frameworks—a *Semantic Gradient framework—*centers on Klein’s (1964) pioneering work. Klein (1964) argued for the existence of what he called a *semantic gradient* within the interference effect. The name, however, is somewhat misleading because as the results of the study showed, the nature of the observed gradient of interference was not exclusively semantic. Klein conducted a between-participants, blocked by stimulus category, Stroop task study where he used four colors as possible responses (red, blue, green, and yellow), and six stimulus categories: nonsense syllables (HJH, EVGJC, BHDR, GSXRQ); rare neutral words (i.e., color-unrelated; SOL, EFT, HELOT, ABJURE); frequent neutral words (PUT, HEART, TAKE, FRIEND); words associatively related to possible responses (LEMON, GRASS, FIRE, SKY); color words representing colors not available as a response (BLACK, GRAY, TAN, PURPLE); and color words representing possible responses (RED, BLUE, GREEN, YELLOW). Klein observed that the magnitude of the obtained interference became gradually stronger as the stimuli became (1) more readable (e.g., nonsense syllables vs. neutral words) and (2) its meaning was more closely related to (response-relevant) colors (e.g., neutral words vs. color-associated words vs. color words). While Klein was the first to discover that different features of stimuli affect the speed of the color-naming response, he was not the first to explicitly propose specific components contributing to Stroop interference. Much later, in Sharma and McKenna (1998), conceptualized the semantic gradient obtained by Klein (1964; see also Fox et al., 1971; Dalrymple-Alford, 1972; Majeres, 1974; Regan, 1978; Li and Bosman, 1996) as reflecting the contribution of various components to the interference effect. The strength of the relation between the meaning of a given stimulus and one of the colors in the experiment was assumed to be captured by a “semantic relatedness” component. The fact that readable stimuli interfered more than non-readable ones was labeled by the authors as a “lexical” component, since to be readable, the stimulus had to be represented in the lexical system.^1^

In the domain of language processing, it is well documented that at least three processes underlie reading: orthographic, lexical, and semantic encoding (e.g., McClelland and Rumelhart, 1981). Orthographic information about the individual letters is represented at the orthographic level. During *orthographic encoding* some of these representations are activated, leading to letter identification. Knowledge as to whether these letters do or do not constitute a real word (e.g., word identification) becomes available through the *lexical encoding*. Letter strings that form real words are also represented lexically. The lexical encoding involves activation of these representations after the word has been (visually) presented. In case of real words, lexical encoding is usually complemented by *semantic encoding*, during which the meaning of the word is accessed.^2^

Apparently, “lexical” and “semantic relatedness” components of Stroop interference proposed by Sharma and McKenna (1998) represent the automaticity of the respective reading sub-stages (e.g., lexical encoding and semantic encoding). Note, however, this is not to say that neutral (i.e., color-unrelated) words that are used to estimate a “lexical” component are only encoded lexically, whereas color-related words that are used to estimate the “semantic relatedness” component are also encoded semantically. Obviously, reading every real word would result in lexical and semantic encoding. However, it is only possible to disentangle between the two, and expose the relative contribution of the automaticity of the semantic encoding to the interference effect by using incongruent color-related words (see a detailed discussion of this issue in the next two paragraphs). In addition, in Klein’s (1964) study, more interference was also observed for nonsense syllables than for colored asterisks (see also Monsell et al., 2001). Based on this finding, we propose that Stroop interference might also have an orthographic component, reflecting the automatic nature of the initial—orthographic encoding—stage of the reading process.

As already mentioned, the *semantic gradient framework* is not the only one addressing the notion of multiple origins of Stroop interference. MacLeod and MacDonald (2000) as well as Goldfarb and Henik (2007) suggested a different perspective that we will refer to as the *two-conflict framework*. In this framework two types of conflict contribute to the Stroop interference. *Task conflict* represents the competition between two possible tasks—the relevant color-naming task and the irrelevant but automatically triggered (by the word stimulus) reading task. The existence of task conflict is supported by neuroimaging and behavioral data. Bench et al. (1993), for example, demonstrated that the anterior cingulate cortex (i.e., ACC) is activated more by incongruent but also congruent color words than by unreadable neutral stimuli (i.e., crosses). Since the ACC is assumed to be involved in conflict monitoring (Carter et al., 1998; Botvinick et al., 2001, 2004), its increased activation by congruent items implies that informationally compatible stimuli may also evoke some kind of conflict. Some researchers noted the ability of various stimuli to trigger the performance of the task they are closely associated with Rogers and Monsell (1995) and Monsell et al. (2001) argued that lexical stimuli such as words, or word-like stimuli (e.g., pronounceable letter-strings) automatically evoke the reading task. In the Stroop task, such an automatic tendency that characterizes congruent but not neutral stimuli might produce a conflict because the stimuli are read instead of being color-named. That is, an increased ACC activation by congruent items is likely an expression of the (task) conflict caused by the automatically performed irrelevant reading task. However, at the behavioral level, reaction times (RTs) to congruent words are in most cases slower than RTs to neutrals—a pattern that one would expect to obtain according to the neuroimaging data. As suggested by Goldfarb and Henik (2007), task conflict that arises in the congruent condition is usually not exposed by behavioral studies due to a very efficient control that operates quickly to eliminate the task conflict. In their, and other studies (Kalanthroff et al., 2013a,b; Entel et al., 2014; Kalanthroff and Henik, 2014) that weakened control by various manipulations, slower RTs to congruent than to neutral stimuli emerged, supporting the notion of the task conflict. Task conflict has also been demonstrated by studies employing Stroop-like task switching paradigms (Aarts et al., 2009; Steinhauser and Hübner, 2009).

When the meaning of the word is related to a color, *informational conflict* arises, enhancing the observed interference. In the color-naming task, the informational conflict can only follow the task conflict and cannot exist by itself because to retrieve the meaning of the word, one should initially start reading it (i.e., perform the irrelevant reading task) (see Levin and Tzelgov, 2014, for a detailed analysis of this issue). Task conflict, in contrast, can exist without informational conflict. When the stimulus is readable but color-unrelated, the extraction of its meaning does not produce informational conflict because color-unrelated meaning does not provide conflicting color information. For instance, the word DOG in red ink would produce task interference because it can be read. However, it would not produce informational interference because it does not belong to the conceptual category of colors (i.e., DOG cannot compete with RED for a response). This notion is critical with regard to the use of the Stroop task in the research on automaticity of reading and its controllability because it emphasizes the importance of task conflict as a marker of automaticity of reading. By contrast, informational conflict is an episodic effect stemming from the dimensional overlap between stimuli and responses (e.g., Kornblum et al., 1990; Zhang and Kornblum, 1998; Zhang et al., 1999). Note that the independence of task conflict from dimensional overlap makes it a “pure” measure (i.e., a marker) of automaticity of the reading process. With this notion in mind, let us introduce the *integrated framework*.

## A Proposed Integrated Framework

In our view, the two frameworks refer to the same idea that can be more elaborated by their integration. Noteworthy, the integration we propose here is not only about suggesting a more consistent taxonomy with regard to the components of the semantic gradient reported by early studies, but about deepening a theoretical understanding of what these components represent. Thus, we believe the part of Stroop interference that expresses the automaticity of reading *per se* arises due to task conflict, and it can be estimated by the orthographic and lexical components of the semantic gradient. The contribution of the informational conflict, which is an episodic amplification of task interference, can be estimated by the semantic relatedness component (Sharma and McKenna, 1998). However, with respect to the latter, we suggest that in order to capture the whole idea of informational conflict, it can be split into two different components. The first component, which we refer to as the *indirect informational conflict*, measures the contribution of the informational conflict caused by color-associated words (e.g., TOMATO). The second component, which we call the *direct informational conflict*, reflects informational interference due to semantic encoding of the color-word stimulus (e.g., RED). The label “indirect” captures the idea that the irrelevant color concept that subsequently competes for response becomes initially activated through its association with another color-associated word, such as the word TOMATO. In contrast, when the stimulus is a color word, the activation of the competing color-concept is “direct,” meaning that it is an outcome of reading the stimulus itself. According to the semantic network model of Collins and Loftus (1975), indirect activation is weaker than the direct one because the activation fades out as it spreads out and is shared between more semantic links. Thus, a color-concept that has been activated indirectly, as in the case of color-associated stimuli, would constitute a weaker competitor in the Stroop task, causing less interference.

The integrated framework suggests a notion particularly important for the research on controllability of the automaticity of reading. This line of research uses modulations of Stroop interference by specific experimental manipulations (e.g., the congruency proportion effects) as an indication of control operation. However, the integrated framework demonstrates that Stroop interference can also be modulated by using a specific stimulus type. The observed interference can be reduced or enhanced depending on the stimulus type that is used in the color-naming task. Employing neutral (i.e., color unrelated) words, for example, would “peel off” the amplification of the interference due to informational conflict, leaving only the contribution of the task conflict.^3^ In this case, the obtained interference effect would be smaller, however, it would be a more precise measure of the automaticity of reading (see the previous paragraph) than the interference effect including informational amplification. Therefore, when investigating the controllability of reading, one should be especially interested in selectively affecting the components reflecting task conflict, which according to the integrated framework, are the orthographic and lexical components. It would be especially interesting to investigate whether the task conflict expressed by each of those components can be controlled. Such a study would shed a light not only on whether reading can be controlled, but on whether such control can be exerted on all reading sub-stages, even the earliest ones, such as the orthographic encoding.

Importantly, in contrast to the previous studies in which the estimation of various components was carried out by performing intuitively more appealing multiple pair-wise comparisons between all stimulus categories used in the experiment, the integrated framework implements a different approach. The disadvantage of the analyses performed in previous studies is that they did not allow for correct estimation of each of the components because of using the same information multiple times. Since according to the integrated framework each of the components has a solid, distinguishable theoretical basis, their estimation should be unique and not contaminated by the information used to estimate other components. For that reason, we used a set of independent contrasts (see **Table 1**), which in our view allows the most adequate and clean estimation of the contribution of each of the four components to the semantic gradient pattern. Hence, within task conflict components, the orthographic component was estimated by contrasting unreadable shapes with the various readable stimuli, color words excluded. The lexical component, representing the modulation of task conflict magnitude by the lexical status of the word, was estimated by comparing minimally readable letter strings with real words, color words excluded. The direct informational conflict component was estimated by contrasting color-word stimuli with all remaining stimuli, whereas indirect informational conflict, representing the modulation of informational conflict by color-related meaning, was estimated by contrasting color-associated words with neutral words. Note, the proposed integrated framework that uses multiple stimulus types and employs a set of independent contrasts allows more stable estimation of some of the underlying interference components. Thus, for example, the estimation of the lexical component, which expresses a difference between readable and unreadable (or minimally readable) material, would be more realistic with regard to the true effect in the general population when calculated according to the proposed framework. This is because, in contrast to what has been usually done,^4^ the “readable” stimuli that are used for its calculation, instead of being represented by only one stimulus category, include a number of similarly readable, yet different stimulus types.

**Table 1:** Contrasts allowing for independent estimation of the semantic gradient components as suggested by the proposed integrated framework.

	Color words (CW)	High frequency color- associated words (CAW H)	Low frequency color- associated words (CAW L)	High frequency neutral words (NeW H)	Low frequency neutral words (NeW L)	Letter strings	Shapes
Orthographic component	0	-1	-1	-1	-1	-1	5
Lexical component	0	-1	-1	-1	-1	4	0
Indirect informational conflict	0	-1	-1	1	1	0	0
Direct informational conflict	6	-1	-1	-1	-1	-1	-1
Lexical frequency effect on task conflict	0	0	0	-1	1	0	0
Lexical frequency effect on informational conflict	0	-1	1	0	0	0	0

*The numbers represent weights used to define each contrast.*

In addition to the estimation of the four main components, we were also interested in accessing the effect of lexical frequency on color naming latencies. Klein’s (1964) observation, replicated later by Fox et al. (1971), was that the RTs produced by “common” neutral words were significantly slower than those produced by “rare” neutral words. The enlarged task interference obtained for high frequency words seems to be consistent with the faster visual recognition of high frequency words reported for word naming and lexical decision tasks (Forster and Chambers, 1973; Monsell et al., 2001). Faster visual recognition is usually attributed to the more efficient lexical encoding/access (Monsell et al., 1989; Murray and Forster, 2004). Thus, it is not surprising that more efficient reading would express itself in larger task interference. Monsell et al. (2001), however, observed slightly shorter color-naming response latencies for the high frequency neutral words than for the low frequency neutral words across the three experiments. In the present study we tested which of these findings can be successfully replicated. Importantly, in addition to neutral words, we also tested the effect of lexical frequency with color-association words in order to investigate whether the effect of lexical frequency can be observed in the domain of informational interference as well. The influence of lexical frequency on the magnitude of task and informational conflicts was assessed separately for neutral words and color-associated words^5^ by contrasting the high and low frequency items in each condition. Note, these comparisons were independent from each other as well as from the rest of the contrasts (see **Table 1**).

To summarize, the goal of the present series of experiments was to put the semantic gradient pattern to another empirical test, while applying the integrated framework to analyze its components. It is our belief that the proposed integrated framework suggests a theoretical and statistical elaboration of the multiple origins of Stroop interference. Along with the empirical investigation reported below and critical review of the literature (see Discussion), it should allow obtaining a clearer sense of what contributes to Stroop interference.

## Experiments 1 and 2

To the best of our knowledge, a semantic gradient has been only reported for English words. The aim of the first two experiments was to evaluate the generality of the semantic gradient pattern phenomenon by testing its existence in Hebrew (Experiment 1) and Russian (Experiment 2). As previously mentioned, the data from these and subsequent experiments were analyzed by carrying out a set of independent contrasts that were aimed at providing independent estimates for each of the four components producing the semantic gradient.

### Method

#### Participants

Twenty-seven (11 females and 16 males) undergraduate students of Ben-Gurion University of the Negev participated in Experiment 1 for course credit. All were native Hebrew speakers, with a mean age of 24.5 years old (*SD* = 2.03). Eighteen undergraduate students (8 females and 10 males) of Ben-Gurion University of the Negev participated in Experiment 2 and were paid 20 NIS. All were native Russian speakers^6^ with a mean age of 26.9 years old (*SD* = 3.45). All reported having normal or corrected-to-normal vision acuity, as well as normal color vision. All participants gave written informed consent in accordance with the Declaration of Helsinki. The experimental protocol was approved by the Ethical Committee of Ben-Gurion University of the Negev.

#### Materials

The stimuli used were Hebrew (Experiment 1) or Russian (Experiment 2) words of the following types^7^: *color words* (adom/ krasnii-RED, kahol/sinii-BLUE, yarok/zelenii-GREEN, tzahov/ jeltii-YELLOW); *high frequency color-associated words* (esh/ ogon-FIRE, agam/nebo–LAKE/SKY, etz/trava-TREE/GRASS, shemesh/solntze-SUN); *low frequency color-associated words* (agvaniya/pomidor-TOMATO, shamaim/djinsi-SKY/DJEANS, esev/lyagushka-GRASS/FROG, tiras/kukuruza-CORN); *high frequency neutral words* (rehov/oficer -STREET/OFFICER, regel/ samolet-LEG/PLANE, mafteah/pis’mo-KEY/LETTER, kvish/ sobaka-ROAD/DOG); *low frequency neutral words* (uga/zontik- CAKE/UMBRELLA, tzipor/golub’-BIRD/DOVE, buba/igla-DOLL/NEEDLE, yareah/koshelek-MOON/WALLET); *letter strings* (shshshsh/ hhhh; ssss/ssss; pppp/oooo; rrrr/rrrr), and *geometric shapes (*rectangle, circle, triangle, and rhombus). The stimuli to be included in each stimulus category were selected based on the norms available in each language—the Russian Frequency Dictionary^8^ developed by Sharoff (2002), and the Word Frequency Database for Printed Hebrew^9^ developed by Frost and Plaut (2005). In addition, the selection was made so that the mean frequency of the two high frequency categories would match as would the mean frequency of the two low frequency categories. Thus, in Experiment 1 the mean frequencies were as follows: 52 and 45 (appearances per million) for high frequency color-associated words and high frequency neutral words, respectively; and 5 and 9 for the same categories but for the low frequency. In Experiment 2, the mean frequencies were 220 and 200 for high frequency color-associated words and neutral words, respectively; and 13 and 14 for low frequency color-associated and neutral words, respectively. In addition, an effort was made to equate all stimulus words for the number of letters as much as it was possible. As for shape stimuli, they were made up of the same number of pixels as the mean number of pixels of the words and letter strings.

The possible ink colors were red, blue, green, and yellow, with the following RGB values: (255, 0, 0) for red; (0, 0, 255) for blue; (0, 128, 0) for green; and (255, 255, 0) for yellow. In order to create only incongruent combinations of stimuli, color words and color-associated words were presented in three of the four possible colors, excluding the color matching their meaning. In contrast, neutral words, letter strings and geometric shapes appeared in all four possible colors. All stimuli appeared in a quasi-randomized order: consecutive trials did not repeat the same word as a word or as a color, and also did not repeat the same color. For example, if in a given trial the word RED appeared in green ink, then in the subsequent trial the stimulus could not be the word RED, GREEN, FIRE, TOMATO, GRASS or TREE, and it could not be printed in red or green ink color.

#### Procedure

A Dell computer with an Intel Pentium Core 2 Duo processor and a 19-inch monitor with a resolution of 1024 × 768 pixels were used to present the stimuli. Participants sat approximately 60 cm from the computer screen. Responses were collected via a high-quality microphone attached to the computer keyboard through a “voice key” device, which allowed RT measurement. In addition, an experimenter coded all responses by typing them on the keyboard. Participants were told not to read the word, but to name its color as accurately and as fast as possible.

The experiment started with seven practice trials followed by two experimental blocks. A 5-min break was given between the blocks. Each of the seven stimulus types was shown 35 times during the block, resulting in total 245 trials per block.

At the beginning of each trial a fixation (white cross) was presented at the center of the screen. After 1,000 ms the fixation was replaced by the target, which remained visible until a response was made or for 3,000 ms. A trial ended with a blank, black display during which the experimenter coded the participant’s response. Trials with technical problems such as laughing or sneezing were coded as technical errors in order to distinguish them from the erroneous responses made by participants.

The design of Experiments 1 and 2 included one within subject variable *of stimulus category* with the following levels: color words, frequent color-associated words, infrequent color-associated words, frequent neutral words, infrequent neutral words, letter strings and shapes. Language frequency, which was relevant only for color-associated and neutral words, was analyzed at the second stage.

### Results

**FIGURE 1:**
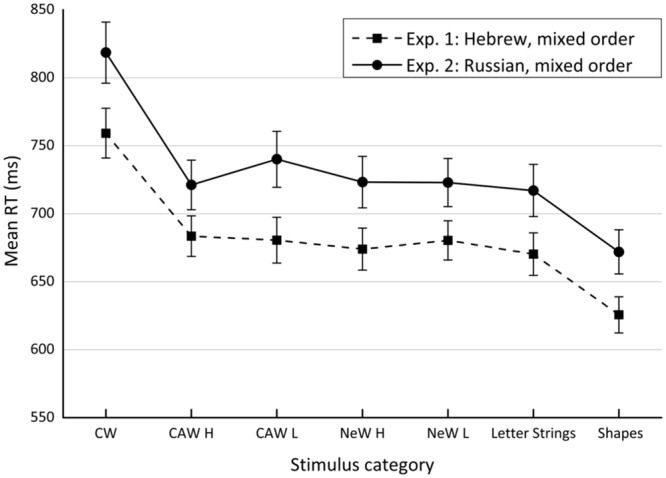
**Reaction time obtained for each stimulus category in Experiments 1 and 2.** CW, incongruent color words; CAW, incongruent color-associated words; NeW, neutral words; H, high frequency; L, low frequency.

Errors, not including technical ones, accounted on average for 1.11% of the trials in Experiment 1 and 2.37% in Experiment 2. All error trials (3.23% in Experiment 1 and 3.43% in Experiment 2) were excluded from the analysis as were the RT outliers (RTs > 2,500 ms and RTs < 300 ms). The mean RTs of correct responses for each participant in each condition were analyzed by a one-way repeated measures analysis of variance (ANOVA). Mean RTs of each experimental condition in the two experiments (as summarized in **Table 2**) are plotted in **Figure 1**. All effects were tested at the significance level (α) of 0.05.

**Table 2:** Mean response times (in milliseconds) obtained in Experiments 1 and 2 for each stimulus category.

	CW	CAW H	CAW L	NeW H	NeW L	Letter strings	Shapes
Experiment 1	759	683	681	674	680	670	626
Experiment 2	818	721	740	723	723	717	672

The results of both experiments revealed a significant main effect of *stimulus category, F*(6,156) = 90.67, *MSE* = 464, $\eta_{p}^{2}$ = 0.77. (Experiment 1) and *F*(6,102) = 34.461, *MSE* = 1,014, $\eta_{p}^{2}$ = 0.7 (Experiment 2). The sum of squares due to the differences among stimulus categories were decomposed by carrying out four orthogonal contrasts (see **Table 1**). These planned comparisons allowed estimating two components of the semantic gradient representing task conflict (i.e., orthographic and lexical components), and two additional components representing informational conflict (i.e., indirect and direct informational conflict components).

#### Planned Comparisons: Experiment 1

Markers of both types of conflict were revealed when Hebrew was used as the experimental language. Thus, the existence of the task conflict was supported by the significant, and quite large, *orthographic component, F*(1,26) = 155.06, *MSE* = 394.4, $\eta_{p}^{2}$ = 0.24, $\eta_{p}^{2}$ = 0.86. The existence of the informational conflict was confirmed by the *direct informational conflict component, F*(1,26) = 160.07, *MSE* = 1,175.6, η^2^ = 0.74, $\eta_{p}^{2}$ = 0.86. However, whereas the magnitude of the task conflict was modulated by the lexical status of the stimuli, showing a significant though relatively small (as evidenced by the $\eta_{p}^{2}$ index) *lexical component, F*(1,26) = 4.83, *MSE* = 382.5, η^2^ = 0.007, $\eta_{p}^{2}$ = 0.16, no indication of modulation of informational conflict by color-related meaning was obtained. That is, there was no significant *indirect informational conflict* component, *F*(1,26) = 2.66, *MSE* = 239.3, *NS*, η^2^ = 0.003, $\eta_{p}^{2}$ = 0.09.

To complete the analysis, the effect of lexical frequency was assessed. In our study lexical frequency was only manipulated for color-associated and neutral words. Since, according to the proposed integrated framework, interference produced by neutral words is only contributed to by task conflict, whereas color-associated words interfere also because of the informational conflict, the effect of frequency produced by both stimulus types was estimated independently. Hence, interference created by frequent neutral words was not significantly different from the interference produced by infrequent neutral words, *F*(1,26) = 2.44, *MSE* = 228.6, *NS*, η^2^ = 0.003, $\eta_{p}^{2}$ = 0.09, implying the task conflict is not enhanced by lexical frequency. The same comparison was conducted for color-associated words, revealing similar results, *F* < 1. Thus, the informational conflict seems not to be affected by lexical frequency.

#### Planned Comparisons: Experiment 2

When Russian was the language of the experiment, the marker of the task conflict (i.e., the *orthographic component*) was successfully replicated, *F*(1,17) = 37.08, *MSE* = 1,133.4, η^2^ = 0.2, $\eta_{p}^{2}$ = 0.69, as was the marker of informational conflict (i.e., the direct informational conflict), *F*(1,17) = 62.99, *MSE* = 2,571.3, η^2^ = 0.77, $\eta_{p}^{2}$ = 0.79. Responses to color-associated words were not slower than the responses to neutral words, *F*(1,17) = 1.46, *MSE* = 694.4, *NS*, η^2^ = 0.005, $\eta_{p}^{2}$ = 0.08, again indicating no modulation of the magnitude of informational conflict by color-related meaning. That is, replicating the result of Experiment 1, no *indirect informational conflict* component was exposed. However, contrary to the previous results, modulation of the task conflict magnitude, as expressed by the *lexical component*, was not obtained, *F*(1,17) = 1.6, *MSE* = 837.5, *NS*, η^2^ = 0.007, $\eta_{p}^{2}$ = 0.09, in the present experiment. In addition, the effect of lexical frequency was estimated by contrasting high frequency and low frequency conditions. Whereas RTs for neutral words were not affected by lexical frequency, *F* < 1, RTs for color-associated words were, *F*(1,17) = 4.77, *MSE* = 672.5, η^2^ = 0.015, $\eta_{p}^{2}$ = 0.22.

### Discussion of Experiments 1 and 2

The data from Experiments 1 and 2 indicate that there are two robust components consistently contributing and almost entirely constituting the interference effect.^10^ These are the markers of the task and informational conflicts (i.e., the orthographic and the direct informational conflict component, respectively), which were easily replicated in two experiments employing different languages. However, the other two components, representing modulation of the magnitude of these conflicts by variables such as the stimulus’ lexical status and semantic distance (i.e., the lexical and indirect informational conflict component, respectively) seem either to appear inconsistently or be hard to obtain. Supportive of this conclusion, both experiments did not succeed in revealing the indirect informational conflict component that was found insignificant and of small size ($\eta_{p}^{2}$ = 0.09 and $\eta_{p}^{2}$ = 0.08 in Experiments 1 and 2, respectively) in both experiments. The same was true for the lexical component ($\eta_{p}^{2}$ = 0.16 and $\eta_{p}^{2}$ = 0.09 in Experiments 1 and 2, respectively), except for it being significant in Experiment 1. Thus, it seems that while these components might occasionally reach a significance level, they are likely to be fragile and to inconsistently contribute to the general interference.

Regarding the effect of lexical frequency, it was only found in the second experiment and only for color-associated words. However, contrary to the findings of Klein (1964) and Fox et al. (1971), performing the color-naming task elicited slower responses to low frequency rather than to high frequency color-associated words. We will further discuss the lexical frequency effect in the Section “General Discussion.”

Yet, one can argue that some differences between the results of the two experiments (e.g., a significance of the lexical component) might partially be due to the fact that Hebrew and Russian belong to different language types. According to the depth-of-orthography hypothesis (Frost, 2006), Hebrew is different from languages like English (and Russian) since the words are written almost solely by consonants and missing the vowels. Thus, reading in Hebrew might proceed differently, or require additional processes (e.g., completing not presented vowel information) than reading in Russian. Hence, it is possible, for example, that in Hebrew all words are initially perceived as letter strings, and considered as words only after the vowel information is completed by additional cognitive processing. If so, then this should lead to a hypothesis inconsistent with present results. Specifically, a “deep-orthography” of the Hebrew language should result in elimination or diminishing of the lexical component in that language, since words are initially perceived as letter strings. However, the two experiments presented reveal the opposite pattern: a significant lexical component in Hebrew, and an insignificant one in Russian. Hence, the differences between the languages used in the present experiments do not seem to be responsible for the obtained pattern.

## Experiment 3

Looking further for the reason why some of the semantic gradient components are not easy to replicate, we reconsidered the methodological details of early studies that reported successful results. In these studies (Klein, 1964; Fox et al., 1971; Sharma and McKenna, 1998), stimuli were presented in a blocked rather than mixed format. Blocking, however, may affect the results by, for example, strengthening the semantic activation of the concepts corresponding to the stimulus words, since each of these repeats itself in a very high temporal proximity. Such temporally proximal repetition of the same words may lead to an accumulation of activation within the orthographic, lexical and/or semantic representations of these words, which in turn can make the relevant interference components visible. Unfortunately, studies that used a mixed presentation format cannot shed light on this issue, since they focused only on particular components (Langer and Rosenberg, 1966; Proctor, 1978; Schmidt and Cheesman, 2005; Risko et al., 2006; Goldfarb and Tzelgov, 2007; Brown, 2011). Therefore, the possibility that the mixed presentation format in our experiments was responsible for the absence of the informational and lexical components from the gradient pattern was tested in Experiment 3. This experiment replicated Sharma and McKenna’s (1998) experimental protocol using Hebrew as the language of the experiment.

### Method

#### Participants

Seventeen undergraduate students (11 females and 6 males) of Ben-Gurion University of the Negev participated in the experiment for course credit. All participants were native Hebrew speakers, had a mean age of 23.5 years old (*SD* = 0.9), and had normal or corrected-to-normal vision acuity, as well as normal color vision. All participants gave written informed consent in accordance with the Declaration of Helsinki. The experimental protocol was approved by the Ethical Committee of Ben-Gurion University of the Negev.

#### Materials

All stimuli and possible ink colors used in present experiment were similar to those used in Experiment 1, except for the following changes. First, the neutral word “yareah” – MOON was replaced with an equally frequent word “rove” – RIFLE. That was done to eliminate a possibly existing association between the word MOON and a yellow color. Second, whereas previously letter strings were generated by using one repeated letter, such as “rrrr”, in the present experiment this was changed and the following letter strings were generated by using a number of various letters: “lgdsh,” “zchma,” “tpsz,” and “tksg.”

#### Procedure

The procedure was almost an exact replication of that in Sharma and McKenna’s (1998) study. The array of all possible combinations of four words of each stimulus category and colors was created in advance. For example, each of the four color words was combined with each of the three incongruent ink colors, thus creating 12 incongruent stimuli. These 12 combinations were repeated five times to produce one experimental block. The stimuli were randomized with the following restrictions: consecutive trials could not repeat the same word and the same color. For instance, in the block of color-word stimuli, if in trial n the word RED appeared in green ink, then in trial n + 1 the words RED and GREEN and the colors green and red could not appear. Importantly, in contrast to the previous two experiments, this procedure, following Sharma and McKenna (1998), ensures that all stimuli appear exactly the same number of times.

In the practice stage, participants performed a 63-trial practice session during which they were familiarized with all possible stimulus categories. After practice, they performed seven experimental blocks—one for each stimulus category—of 60 trials each, resulting in 420 trials in total. The blocks (except for the one with shapes that was always presented as a first block) were counterbalanced across participants by applying a Latin square technique. Additionally, similar to the original study by Sharma and McKenna (1998), before each block participants were informed what category of stimuli (words, letter strings, or shapes) was going to appear next.

As in the original Sharma and McKenna’s (1998) study and in contrast to the first two experiments reported above, an inter-stimulus interval of two seconds was introduced. That is, after a participant made a response and it was coded by the experimenter, there was a 2-s period without any stimulation.

As in the two previous experiments, the design included one within-subject factor: *stimulus category* (color words, high frequency color-associated words, low frequency color-associated words, high frequency neutral words, low frequency neutral words, letter strings and shapes), with the effects of frequency being analyzed in the second stage. Another between-participant factor, employed for reasons of experimental control, was the *order of blocks* (A, B, C, D, E, F).

### Results

**FIGURE 2:**
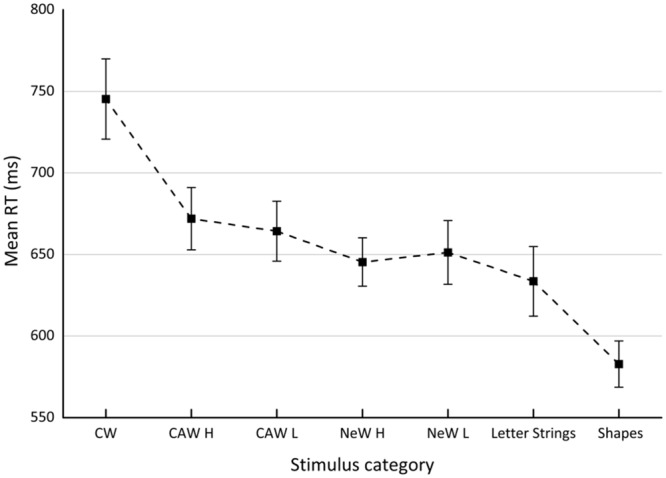
**Reaction time obtained for each stimulus category in Experiment 3.** CW, incongruent color words; CAW, incongruent color-associated words; NeW, neutral words; H, high frequency; L, low frequency.

Errors, excluding technical ones, accounted for an average of 2.24% of the trials. All errors (4.82%) as well as the RT outliers (RTs > 2,500 ms and RTs < 300 ms) were excluded from the analysis. Mean RTs for each condition as summarized in **Table 3** are graphically presented in **Figure 2**. All effects were tested at the significance level (α) of 0.05. A two-way repeated measures ANOVA revealed a significant effect for stimulus category, *F*(6,66) = 30.73, *MSE* = 1,298, $\eta_{p}^{2}$ = 0.73, which did not interact with the order of blocks, *F*(30,66) < 1. There was no main effect of order of blocks variable as well, *F*(5,11) < 1.

**Table 3:** Mean response times (in milliseconds) obtained in Experiment 3 for each stimulus category.

	CW	CAW H	CAW L	NeW H	NeW L	Letter strings	Shapes
Experiment 3	747	672	663	646	652	634	583

As was found previously, planned comparisons revealed a strong marker of the task conflict as measured by *the orthographic component, F*(1,11) = 67.4, *MSE* = 1,016.8, η^2^ = 0.29, $\eta_{p}^{2}$ = 0.86, as well as a marker of the informational conflict [i.e., *direct informational conflict, F*(1,11) = 53.9, *MSE* = 2,896.5, η^2^ = 0.65, $\eta_{p}^{2}$ = 0.83]. Regarding the modulation effects, similar to the previous two experiments, no evidence for contribution of *indirect informational conflict* was found, *F*(1,11) = 3.67, *MSE* = 1,507.6, *NS*, η^2^ = 0.02, $\eta_{p}^{2}$ = 0.25. Surprisingly, after being absent in Experiment 2, the *lexical component* showed up again in the present experiment, *F*(1,11) = 7.1, *MSE* = 1,148.3, η^2^ = 0.03, $\eta_{p}^{2}$ = 0.39. The effect of lexical frequency was not observed for neutral words, *F* < 1, or for color-associated words, *F*(1,11) = 1.3, *MSE* = 458, *NS*, η^2^ = 0.003, $\eta_{p}^{2}$ = 0.11.

### Discussion

The results of this study imply that blocking stimulus categories might affect the modulation effects of the informational and task conflict. This conclusion is based on the observation of the lexical component, which was evident in Experiment 1, disappeared in Experiment 2, and was detected once again in the present experiment where the various stimuli categories were blocked. Importantly, compared to previous experiments, in the present one the effect size of the task conflict modulation effect was quite impressive, $\eta_{p}^{2}$ = 0.39. The same can also be said about the non-significant modulation effect of the informational conflict. The effect size of the indirect informational conflict in the present experiment was $\eta_{p}^{2}$ = 0.25, whereas in the first and second experiments it was only 0.09 and 0.08, respectively. However, to reach such a conclusion one needs to manipulate the presentation format within the same experiment to compare directly the pattern obtained with mixed versus blocked format. That was done in Experiment 4.

In addition, it is possible that the absence of the difference between color-associated words and neutral words (in all three experiments the indirect informational conflict component never reached significance) could be due to the fact that the color-associated words used in our experiments were not associated strongly enough with the related color.^11^ Such a weak association could make these words behave like the neutral ones. In addition, while controlling for lexical frequency, number of letters, and concreteness, we were not able to control for other important parameters of words, namely, the number of semantic neighbors (i.e., all associative connections of a given concept). Nonetheless, this factor can affect the results. The more semantic neighbors a given color-associated word (e.g., FIRE) has, the lower the activation that would propagate to each of its neighbors, including the related color concept (e.g., RED) (Collins and Loftus, 1975; Buchanan et al., 2001). In this case, even if the association between FIRE and RED was strong, only a small interference, if any, would be obtained with these words as Stroop stimuli. To test if one of those alternatives could explain the insignificance of the indirect informational conflict component, in Experiment 4 the strength of associations (between color-associates and color concepts they are related to) was manipulated.

## Experiment 4

The aim of the present experiment was twofold. First, we wanted to test directly whether presentation format (mixed vs. blocked) would affect the magnitude of the semantic gradient components, especially those that were shown to appear inconsistently (i.e., the lexical and indirect informational conflict components). Second, we were interested to investigate the possibility that the insignificance of the indirect informational conflict component in present series of experiments might be due to relatively weak associations existing between the words that were selected as color-associates and respective colors. The latter was done by exposing half of the participants to color pictures of color-associated words (e.g., a picture of a red tomato) during the pre-test stage. It was expected that this manipulation would strengthen the (naturally) existing association between words and associated colors, which would help in revealing an indirect informational conflict component. Moreover, it is likely that this manipulation would add a strong visual representation to verbal information presented by the words (Sadoski and Paivio, 2004). Such visual representation is able to enhance the activation of the long-term semantic representation of the word after the latter has been read, which may help to overcome the problem of multiple semantic neighbors by means of increased initial activation.

### Method

#### Participants

Seventy-two undergraduate students (60 females and 12 males) of Ben-Gurion University of the Negev participated in the experiment for course credit. The mean age of the participants was 23.1 years old (*SD* = 0.87), all were native Hebrew speakers and all reported having normal or corrected-to-normal vision acuity, as well as normal color vision. No one reported having a learning disability or attention deficit/hyperactivity disorder. All participants gave written informed consent in accordance with the Declaration of Helsinki. The experimental protocol was approved by the Ethical Committee of Ben-Gurion University of the Negev.

#### Materials

The stimuli used in the Stroop task were identical in all respects to those used in Experiment 3. However, in contrast to the previous experiments, before performing the usual Stroop task, half of the participants were exposed to pictures of the color-associated and neutral words. Pictures of color-associated words were shown in color (such as a picture of a red tomato or a blue sky), whereas pictures of neutral words were in black and white.

#### Procedure

The experiment consisted of three stages: a picture recognizing stage, a Stroop task stage, and a memory test stage. Half of the participants performed all three stages, whereas the other half performed only the Stroop task stage. In the *picture recognizing stage*, participants were presented with the pictures of the words that were later used as stimuli in the Stroop task. They were asked to indicate what they saw in the picture by ticking the box with the relevant answer. In each trial, three possible answers were presented below the picture. The position of the correct answer varied in a random fashion. To allow strengthening the existing color association, each picture was shown four times during this stage, resulting in 64 trials. Participants were informed that at the end of the experiment they would be asked to perform a memory test, checking how well they remembered the pictures that they were currently seeing. They were told that there was no need to hurry and respond quickly, and that they could take as much time as needed to look at each picture, memorizing its details. In the *Stroop task stage*, participants performed a Stroop task during which they were presented with all possible stimulus categories. Importantly, for half of the participants the stimulus categories were mixed, and for the other half they were blocked. In addition to manipulating presentation format, two additional changes were applied to the experimental protocol of Experiment 3. Each block was enlarged from 60 to 72 trials. In contrast, the duration of the inter-stimulus-interval was shortened from 2,000 to 1,500 ms. All other details of the Stroop task procedure remained unchanged. Finally, in the *memory test stage*, the memory of the pictures presented was assessed. Participants were shown 30 words and asked to determine if they saw the picture of those words during the picture recognizing stage. Pictures of 16 words (eight color-associated and eight neutral) were actually presented at the beginning of the experiment. The remaining 14 words were not previously shown to the participants as pictures, but they were shown as alternative (incorrect) responses to the pictures (see **Table 4**). After each response, the participants were also asked how confident they were about their answer: “very sure,” “sure,” or “not sure.” In addition, if the participants confirmed seeing the picture of the presented word, they were asked if that picture was presented in color or in black and white.^12^

**Table 4:** Color pictures of color-associated words and black and white pictures of neutral words that were used as stimuli in the pre-test stage of Experiment 4.

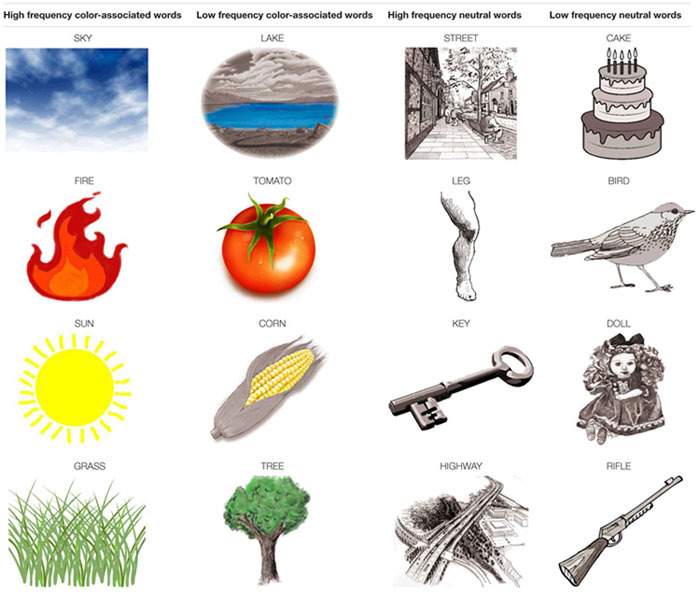

The design of the experiment included two between-participants variables: *presentation type* (mixed/blocked) and *color-association strength* (primed/unprimed). The only within-subject variable was *stimulus category* (color words, high frequency color-associated words, low frequency color-associated words, high frequency neutral words, low frequency neutral words, letter strings and shapes). As previously, language frequency was initially treated as part of the stimulus category variable, whereas its effect was planned to be later assessed by planned comparisons.

### Results

**FIGURE 3:**
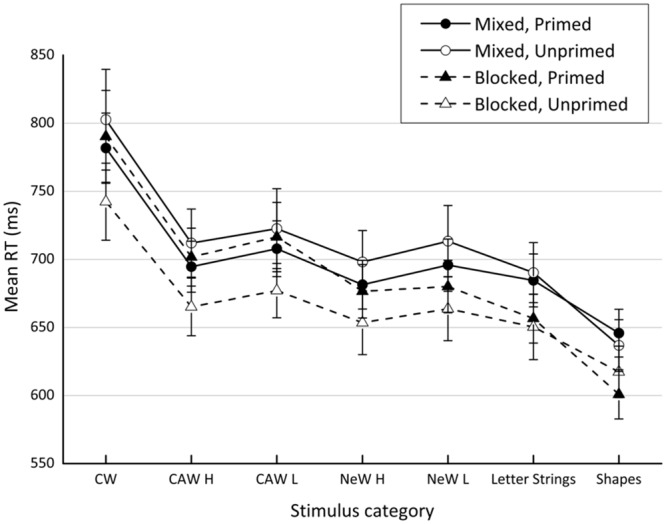
**Results of Experiment 4.** The figure presents mean reaction time obtained for each stimulus category in each presentation format (mixed/blocked) for participants who were exposed to pictures and those who weren’t (primed/unprimed) in the pre-test stage. CW, incongruent color words; CAW, incongruent color-associated words; NeW, neutral words; H, high frequency; L, low frequency.

Non-technical errors accounted on average for 2.26% of the trials. All error trials (6.4%) as well as the RT outliers (RTs > 2,500 ms and RTs < 300 ms) were excluded from the analysis. Mean RTs for each condition as summarized in **Table 5** are plotted in **Figure 3**. All effects were tested at the significance level (α) of 0.05.

**Table 5:** Mean response times (in milliseconds) for each stimulus category in each presentation format and color association strength condition of Experiment 4.

Experiment 4	CW	CAW H	CAW L	NeW H	NeW L	Letter strings	Shapes
Blocked, Primed	790	701	716	676	680	656	601
Blocked, Unprimed	742	665	677	653	664	650	617
Mixed, Primed	782	695	708	681	696	684	646
Mixed, Unprimed	801	712	723	698	713	690	637

A repeated measures ANOVA revealed a significant three-way interaction between presentation format*color-association strength*stimulus category, *F*(6,408) = 3.36, *MSE* = 1,357, $\eta_{p}^{2}$ = 0.05. As a first step in decomposing this interaction, the simple two-way interaction between color-association strength*stimulus category was tested for significance at each level of the presentation format variable. Thus, when the stimuli were blocked^13^ the significant interaction between color-association strength and stimulus category was obtained, *F*(6,204) = 2.4, *MSE* = 4,422, $\eta_{p}^{2}$ = 0.07; that is, the pattern observed for stimulus category was affected by the presentation of pictures. When the associations between color-associated words and colors were strengthened by exposing participants to pictures of these words, all semantic gradient components were present, including those that appeared inconsistently (or never appeared) before. That is, there were four significant components: an *indirect informational conflict component, F*(1,17) = 21.6, *MSE* = 804, η^2^ = 0.05, $\eta_{p}^{2}$ = 0.55; a *lexical component, F*(1,17) = 19.6, *MSE* = 1,013, η^2^ = 0.05, $\eta_{p}^{2}$ = 0.53; an *orthographic component, F*(1,17) = 57.6, *MSE* = 1,900, η^2^ = 0.3, $\eta_{p}^{2}$ = 0.77; and a *direct informational conflict component, F*(1,17) = 31.4, *MSE* = 6,886, η^2^ = 0.59, $\eta_{p}^{2}$ = 0.65. Lexical frequency did not affect the response times to color-associative words, *F*(1,17) = 1.5, *MSE* = 1,281, *NS*, η^2^ = 0.005, η^2^_p_ = 0.08, or to neutral words, *F* < 1. However, when no pictures were shown to participants prior to the Stroop task, only two components were demonstrated. Replicating the results of previous experiments, these were the marker of the task conflict—*the orthographic component, F*(1,17) = 13.1, *MSE* = 2,275, η^2^ = 0.19, $\eta_{p}^{2}$ = 0.44—and marker of the informational contribution—the *direct informational conflict component, F*(1,17) = 47.4, *MSE* = 2,515, η^2^ = 0.76, $\eta_{p}^{2}$ = 0.74. The *indirect informational conflict component* was not observed, *F*(1,17) = 1.8, *MSE* = 1,576, *NS*, η^2^ = 0.02, $\eta_{p}^{2}$ = 0.09, as was the case for the *lexical component, F*(1,17) = 3.3, *MSE* = 913, *NS*, η^2^ = 0.02, $\eta_{p}^{2}$ = 0.16. Once again, lexical frequency was not found to affect the color-associated words, *F*(1,17) = 2.4, *MSE* = 535, *NS*, η^2^ = 0.008, $\eta_{p}^{2}$ = 0.13, or the neutral words, *F*(1,17) = 1.5, *MSE* = 620, *NS*, η^2^ = 0.005, $\eta_{p}^{2}$ = 0.08.

As for the mixed condition, the two-way color-association strength^∗^stimulus category interaction failed to reach significance, *F*(6,204) = 1.09, *MSE* = 976, $\eta_{p}^{2}$ = 0.03. Hence, in the mixed condition the same stimulus category pattern was obtained whether the participants were exposed to pictures of the words in the pre-test stage or not. Surprisingly though, this pattern included *all* four components, which according to the proposed integrated framework, contribute to the semantic gradient. Four orthogonal contrasts conducted across the levels of the color-association strength variable revealed a significant *indirect informational conflict* component, *F*(1,34) = 14.2, *MSE* = 368.7, η^2^ = 0.01, $\eta_{p}^{2}$ = 0.29, as well as significant *lexical component, F*(1,34) = 20, *MSE* = 360.2, η^2^ = 0.02, $\eta_{p}^{2}$ = 0.37). In addition to these somewhat elusive (based on the data from the previous experiments) components that suddenly appeared in the present experiment, more robust markers of both types of conflict were replicated once again. The task conflict contribution was expressed by the significant *orthographic component, F*(1,34) = 152.6, *MSE* = 678.5, η^2^ = 0.23, $\eta_{p}^{2}$ = 0.82, whereas the informational conflict was marked by its *direct component, F*(1,34) = 97.4, *MSE* = 3,292, η^2^ = 0.72, $\eta_{p}^{2}$ = 0.74. Surprisingly, a significant effect of lexical frequency was observed for color-associative words, *F*(1,34) = 8.3, *MSE* = 306, η^2^ = 0.03, $\eta_{p}^{2}$ = 0.20, as well as for neutral words, *F*(1,34) = 11.6, *MSE* = 342, η^2^ = 0.05, $\eta_{p}^{2}$ = 0.25. In both conditions these were the less frequent words that produced slower responses.

### Discussion

At the first glance, the results obtained in the blocked presentation condition seem to be consistent with the hypothesis suggesting that stronger associations between color-associated words and related colors are needed to reveal the indirect informational conflict component. When these associations were enhanced by presenting participants with color pictures of the stimulus words, the previously missing indirect informational conflict component was successfully exposed. In addition, the same condition also showed a significant lexical component, which appeared inconsistently in the previous experiments. Whereas the latter result may be a consequence of strengthening the visual code of the word stimuli (at the pre-test stage the black-and-white pictures of neutral words were presented along with the color pictures of color-associated words) or a result of blocking the stimuli, it also may be unrelated to any of the manipulations used in the experiment as it seemed to be in the previous experiments. In fact, the elusive nature of the lexical component as well as of the indirect informational conflict component seems likely, especially in light of the results obtained in the mixed presentation condition, which demonstrated all components contributed to a general interference effect, including lexical and indirect informational conflict components. These results are quite surprising since, unlike in the blocked condition, in the mixed condition all the semantic gradient components appeared regardless of the experimental manipulation of the association strength. However, in the previous experiments when the association strength was not manipulated and stimuli were presented in a mixed fashion, the entire semantic gradient was never exposed. These puzzling results as well as the effects of lexical frequency are further discussed in Section “General Discussion.”

## General Discussion

The results of the last experiment further support the conclusion provided by the three previous experiments, suggesting that the semantic gradient is almost entirely made up of two robust and easily replicated components. The orthographic component expressing the task conflict contribution and the direct informational conflict component expressing the informational conflict contribution, were consistently demonstrated by each experiment in the present series. The other two components of the semantic gradient only occasionally modulated performance and the conditions for their appearance were difficult to specify (see **Table 6**). Thus, the lexical component that modulates task conflict by the lexical status of the stimulus, appeared in the first experiment (Hebrew, mixed presentation), but contrary to the depth-of-orthography hypothesis, disappeared in the second experiment (Russian, mixed presentation). The lexical component was obtained in Experiment 3, where Sharma and McKenna’s (1998) experimental protocol was carefully replicated. However, proving its elusive nature, the lexical component appeared inconsistently across the various conditions in the last experiment. Thus, it was observed in both mixed conditions (i.e., across the association strength levels). However, in the blocked conditions the lexical component was obtained only when the color-word associations were strengthened by presenting participants with color pictures of these words. Since the manipulation of the strength of association is essentially irrelevant to the lexical component and was aimed at exposing the indirect informational conflict component, it is not clear why the lexical component did not consistently appear in both blocked conditions. Moreover, there were no methodological or other differences between the blocked condition employed in Experiment 3 and the parallel (blocked/unprimed) condition of Experiment 4, and yet the lexical component was only demonstrated by the former^14^.

**Table 6:** Summary of effect sizes ($\eta_{p}^{2}$) obtained for each of the semantic gradient components.

		Orthographic component	Lexical component	Indirect informational conflict	Direct informational conflict
Experiment 1	Hebrew, mixed	$\eta_{p}^{2}$ = 0.86^∗^	$\eta_{p}^{2}$ = 0.16^∗^	$\eta_{p}^{2}$ = 0.09	$\eta_{p}^{2}$ = 0.86^∗^
Experiment 2	Russian, mixed	$\eta_{p}^{2}$ = 0.69^∗^	$\eta_{p}^{2}$ = 0.09	$\eta_{p}^{2}$ = 0.08	$\eta_{p}^{2}$ = 0.79^∗^
Experiment 3	Hebrew blocked	$\eta_{p}^{2}$ = 0.86^∗^	$\eta_{p}^{2}$ = 0.39^∗^	$\eta_{p}^{2}$ = 0.25	$\eta_{p}^{2}$ = 0.83^∗^
Experiment 4	Blocked, primed	$\eta_{p}^{2}$ = 0.77^∗^	$\eta_{p}^{2}$ = 0.53^∗^	$\eta_{p}^{2}$ = 0.55^∗^	$\eta_{p}^{2}$ = 0.65^∗^
	Blocked, unprimed	$\eta_{p}^{2}$ = 0.44^∗^	$\eta_{p}^{2}$ = 0.16	$\eta_{p}^{2}$ = 0.09	$\eta_{p}^{2}$ = 0.74^∗^
	Mixed, across priming conditions	$\eta_{p}^{2}$ = 0.82^∗^	$\eta_{p}^{2}$ = 0.37^∗^	$\eta_{p}^{2}$ = 0.29^∗^	$\eta_{p}^{2}$ = 0.74^∗^
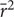 _contrast_		*0.77*	*0.28*	*0.32*	*0.77*

*^∗^Significant results. 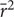
_*contrast*_ (Rosnow et al., 2000; Rosnow and Rosenthal, 2002, 2009) represents the combined effect size across the four experiments.*

The indirect informational conflict component, which represents modulation of the informational conflict magnitude due to the color-related meaning of the stimulus, did not appear in any but the last experiment, and even there it seems not to have been related to the manipulation employed in order to reveal it. It was expected to be present either when the color-word associations were strengthened by a priming manipulation, or (and) when the stimuli were blocked by type. However, contrary to expectations, the indirect informational conflict component was unexpectedly found in both mixed conditions, whether the associations were enhanced or not. In addition, it was also evident in the blocked conditions with strong associations. As will shortly be more broadly discussed, such unpredictable behavior of this component, as well as its clear unrelatedness to experimental manipulations, demonstrated by this and previous experiments, implies that one cannot expect to consistently observe this component as being part of the semantic gradient.

As to the lexical frequency effect, it was observed to modulate the task interference produced by neutral words only once—in the mixed condition of Experiment 4. Even then, similar to Monsell et al.’s (2001) findings, the effect was in the opposite direction to that reported by Klein (1964) and Fox et al. (1971): high frequency words produced faster responses than low frequency words. The same direction of the effect was obtained for informational interference of the color-associated words. The latter effect, however, seems to be more persistent, and although it reached significance level only twice—in Experiments 2 and 4 (mixed condition)—it demonstrated a larger effect size than its neutral words counterpart in all but the first experiment. That is, according to our results, the effect of word frequency seems to be more pronounced for color-associated than neutral words. However, since the reliability of this effect appeared to be limited, we prefer to regard this interpretation as speculative. Careful investigation of this issue that would take into account a possible confounding effect of other properties of the stimulus words (e.g., syntactic class, number of lexical neighbors; see Monsell et al., 2001), as well as the counteracting effects of word repetitions (for review see Monsell, 1991), might be a fruitful direction to pursue in future studies.

Our study is not unique with respect to the inconsistency of contribution of the lexical and indirect informational conflict components to the interference effect. Interpreting the results of previous studies related to the semantic gradient phenomenon in terms of components representing two different conflict types, clearly shows that along with the studies demonstrating the existence of the lexical and indirect informational conflict components, there are also some that failed to find them. Thus, in Keele’s (1972) study no lexical component as measured by the difference between the scrambled letters condition and the neutral words conditions was found. Surprisingly, any other marker of the task conflict was absent as well; the control condition including Gibson forms elicited the same RTs as did the letter strings and neutral words conditions. This, however, may be related to the manual response modality used in that study, which usually allows for less interference to be observed (for a review see MacLeod, 1991) and as such makes it difficult to reveal all of its components (Sharma and McKenna, 1998). Following Keele’s study, Hintzman et al. (1972) conducted a similar experiment but used a vocal response. In addition, unlike Klein’s (1964) study that manipulated stimulus types between participants and used a blocked, card design, Hintzman et al. (1972) used a within-subject, mixed design, with each stimulus presented one at a time. Thus, the latter study resembled the first and second experiments of the present study, where all participants were exposed to all stimulus types when they were presented in a mixed order, one stimulus at a time. The results showed that the RTs to neutral words did not differ from the RTs elicited by letter strings, demonstrating no lexical component. Similar results were obtained in the study of Glaser and Glaser (1989, Experiment 5). In that experiment the authors attempted to replicate the semantic gradient with separated, instead of integrated, Stroop stimuli (words of various types superimposed on colored rectangles) and found no significant difference between the *word-control* (i.e., the distracter was a color-unrelated word) and *letter-control* conditions (i.e., the distracter was a letter string).

As for the indirect informational conflict component, Glaser and Glaser (1989) reported a significant 14 ms difference between the *far incongruent* condition (i.e., the distracter was a color-associated word) and the *word-control*, as well as *letter-control* conditions. However, since no effect size index was provided, it is hard to know how large and stable this effect was. More recent works represent the same picture showing somewhat inconclusive results with respect to the indirect informational conflict component. Risko et al. (2006) focused exclusively on the difference between color-associated words and neutral words in their study. They found that color-associated words that were relevant to colors in the response set elicited more interference than those which were irrelevant to the response set. The authors also claimed, based on an additional, though dependent, comparison, that even color-associated words that were irrelevant to the response set were more interfering than neutral words were. However, the aforementioned difference was only 11 ms in the vocal response experiment, and 7 ms in the manual response experiment. Since no effect size measures were provided, there is room to assume that even if the indirect informational conflict component found in this study existed, it was very small and probably highly unstable. Another study, by Schmidt and Cheesman (2005), investigated an unrelated question of dissociation between stimulus–stimulus and response–response effects in the Stroop task. However, the design of that study included manipulation of the stimulus type, which makes it relevant to the present discussion. They employed color words, color-associated words and direction words (e.g., left), and responses were made manually by pressing two different keys. Direction words, however, may be considered to be a category of neutral words (i.e., color-unrelated), similar to, for example, animal words (e.g., dog) used by Tzelgov et al. (1992) as a neutral condition. As such, in our view, this design allows testing the indirect informational conflict component, even though it was not originally designed for doing so. In order to recalculate the mean RTs of the relevant conditions, we ignored the congruent stimuli and calculated the RTs to incongruent color words, incongruent color-associated words and direction words by averaging the provided RTs across the levels of response type variable, which we were not concerned with. The RTs we obtained as a result of this analysis were 568 ms for color words, 550 ms for color-associated words and 544 ms for direction (neutral) words. That is, the indirect informational conflict component in this study was indicated by a 6 ms difference between color-associated words and neutral words. Even though a gross value like this cannot be used as a measure of the effect size, such an extremely small difference is likely to imply that the contribution of the indirect informational conflict to the interference effect was negligible and probably unstable.

As can be seen from the critical review of the literature, there is an abundance of studies that, like the present one, did not succeed in revealing the components of the semantic gradient reported by earlier studies (Klein, 1964; Fox et al., 1971; Dalrymple-Alford, 1972; Majeres, 1974; Regan, 1978; Li and Bosman, 1996; Sharma and McKenna, 1998). Specifically, the lexical and indirect informational conflict components were shown to be very fragile—they appeared inconsistently and seemed to be of a very small size even when estimated outside the semantic gradient framework. However, in our view the inconsistency of observing the aforementioned components is more likely to be related to a relatively small size of these effects in the population rather than to the expression of their complete inexistence. The analysis of the Bayes factor, as summarized in **Table 7**, supports this conclusion. Of the six times each of these components were estimated across the four experiments, only once was the evidence supportive of a NULL (i.e., non-existent) effect for the lexical component, and even this evidence was categorized as “anecdotal” based on Wetzels et al.’s (2011) categorization. Regarding the indirect informational conflict, the Bayes factor was supportive of a NULL effect in three of the six comparisons. However, all this evidence was “anecdotal” as well, meaning that “inexistence” is not more probable that the “existence” of the effect given the data. In contrast, in the other three comparisons the Bayes factor showed clear support for the existence of indirect informational conflict, which varied from anecdotal to decisive.

**Table 7:** Bayes factor of each contrast performed to estimate a specific component.

	Experiment 1 Hebrew, mixed	Experiment 2 Russian, mixed	Experiment 3 Hebrew blocked	Experiment 4 Blocked, primed	Experiment 4 Blocked, unprimed	Experiment 4 Mixed, across priming conditions
Lexical component	0.5 Anecdotal evidence for H_1_	1.9 Anecdotal evidence for H_0_	0.2 Substantial evidence for H_1_	0.007 Decisive evidence for H_1_	0.94 Anecdotal evidence for H_1_	0.002 Decisive evidence for H_1_
Indirect informational conflict	1.4 Anecdotal evidence for H_0_	2 Anecdotal evidence for H_0_	0.7 Anecdotal evidence for H_1_	0.005 Decisive evidence for H_1_	1.85 Anecdotal evidence for H_0_	0.02 Very strong evidence for H_1_

*The Bayes factor was calculated based on Campbell and Thompson (2012) as the relation between (posterior) probabilities of obtaining each one of the hypotheses—H_1_ representing an effect, and H_0_ representing the NULL effect—given the data. The text provides an interpretation of a given value according to the categorization suggested by Wetzels et al. (2011).*

Hence, following that the NULL hypothesis did not receive substantial support in either of the performed experiments, whereas the alternative hypothesis sometimes did, the general conclusion from this study would be that the lexical and indirect informational conflict components do contribute to the semantic gradient, however, their contribution is much smaller than the contribution of other components. This interpretation is readily supported by the results of the meta-analysis of the data obtained across the four experiments in the present study. As indexed by the 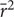
_contrast_ values reported in **Table 6**, the lexical and indirect informational conflict components appear to be as much as two times smaller in magnitude, than the orthographic and direct informational conflict components. The latter two, however, have proved to be very robust: they were exposed in each of the conducted experiments and consistently demonstrated quite large size (see **Table 6**). Thus, it can be summarized that out of the two distinct components representing each conflict type, one can be defined as the *major contributor* to Stroop interference, being large and robust (i.e., the orthographic component in the case of task conflict, and the direct informational conflict component in the case of informational conflict), whereas the second can be considered a *minor contributor* (i.e., lexical component representing task conflict, and indirect informational conflict representing the informational conflict), due to its small size and inconsistent appearance.

We believe our results showing that some components of the sematic gradient are small and unstable, along with the reports in the literature discussed above, call for reconsideration of the semantic gradient as representing a *linear* increase in interference due to various stimulus features. Recognition that the semantic gradient is not as much of a “gradient” as it was thought to be, might have an important contribution, especially to studies that base their predictions and experimental manipulations upon replication of the findings of early studies such as those by Klein (1964) and Sharma and McKenna (1998). Expecting some of the semantic gradient components to be as large and as robust as others, when in fact they are small and unstable, may misdirect and even fail future studies. Taking into account possible differences in the size of the components may help such studies to be planned accordingly by creating an experimental situation in which those components are more likely to be exposed. It is worth noting, however, that although the present results refute the notion of linearity of the interference accumulation, they do not reject the very existence of the semantic gradient. The core implication of this phenomenon, namely, the magnification of interference due to such features as the level of stimulus readability and the level of its color-relatedness, is supported by the present data.

The notion that each type of conflict has a major and a minor representation might have a potential to contribute to the understanding of cognitive control in the automatic reading process. As we have recently discussed (Levin and Tzelgov, 2014), the control of a “pure” task conflict, when it is not amplified by informational conflict, has yet to be explored. Such an exploration requires using color-unrelated stimuli when manipulating cognitive control. The present results can offer some guidance with respect to selection of specific stimuli as well as to the prediction of a possible outcome. For example, according to our findings, the magnitude of the task conflict produced by real words does not consistently differ from that produced by letter strings; however, the task conflict produced by both stimulus types differs sharply from that produced by stimuli that are totally unreadable shapes. Hence, when selecting stimuli in order to efficiently manipulate the magnitude of the “pure” task conflict, one might want to use both letter strings and real words as “conflicting” stimuli instead of using only one of them, to achieve a more stable estimate of the task conflict. Regarding the indirect informational conflict component, the present results imply that the amplification of the task conflict by the informational conflict may be prone to disappearance when it proceeds indirectly, through color association. This should also be considered when planning and interpreting experiments aimed at investigating the selective control mechanism(s) of task and informational conflicts. In this context, there is another important issue to point out—the use of congruent items. In fact, it may even explain why some of the aforementioned studies might be more successful in revealing the elusive lexical component. As suggested by MacLeod and MacDonald (2000), congruent items may be more promptly read by the participants because their reading provides a correct response (an inadvertent reading hypothesis). Hence, introducing these stimuli in the list may induce a stronger general tendency to read the encountered stimulus (for a similar discussion see Goldfarb and Henik, 2013). That is, using congruent items might - Funding query have a direct effect on the magnitude of the task conflict, resulting in enhanced lexical and orthographic components. Future study is needed to provide a direct investigation of this issue.

## Author Contributions

YL participated in planning designing running the experiments, analyzing the data and preparing the manuscript. JT participated in planning the experiments and preparing the manuscript.

## Conflict of Interest Statement

The authors declare that the research was conducted in the absence of any commercial or financial relationships that could be construed as a potential conflict of interest.

## Footnotes

1 Irrelevant for present discussion, the authors also reported a “semantic relevance” component, which was a difference between the stimuli representing actual colors in the experiment and stimuli representing other colors than these used in the experiment.

2 Note that in order to avoid terminological confusion, the present work defines semantic representation as referring to the meaning of a word.

3 Although we were not interested in doing so in the present study, the contributions of the response and informational conflict can be disentangled in a similar way, by introducing color words that do not constitute a valid response into the design (non-response color-words; Klein, 1964; Sharma and McKenna, 1998).

4 Either as part of conducting pair-wise comparisons or because the design includes only two stimulus types, most of the studies contrast between the two exclusive stimulus categories to estimate the relevant interference component.

5 It was not tested under a color word condition because it would inevitably introduce a response set membership confound (for review see MacLeod, 1991).

6 However, the participants were also fluent in Hebrew because they lived in Israel for a number of years. Since it was impossible to recruit completely monolingual native Russian speakers, an effort was made to select unbalanced bilinguals who still considered Russian as their dominant language. Accordingly, the criteria for selecting participants were as follows: (1) Russian had to be a participant’s mother tongue, meaning that the participant listened to this language since childhood and learned it as the first language; (2) a participant had to have lived in Israel for not more than 15 years; and (3) only volunteers who preferred to speak, read books, and watch movies in Russian on a daily basis. Importantly, all interactions between the participant and the experimenter were carried out in Russian in order to create a nearly monolingual atmosphere.

7 Specific words included in each category are presented in parenthesis; the first word represents the word in Hebrew, the second word, appearing after the slash, represents the word in Russian, and the English word/s, appearing after the dash, represent/s the respective translation of the Hebrew and Russian words to English (e.g., agam (Hebrew)/nebo (Russian)–LAKE (Hebrew word translation)/SKY (Russian word translation).

8 http://bokrcorpora.narod.ru/frqlist/frqlist-en.html

9 http://word-freq.mscc.huji.ac.il/index.html

10 As evidenced by the sum of the η^2^ indexes, the orthographic and direct informational conflict components accounted for 98% in Experiment 1 and 97% in Experiment 2 of the systematic variance existing in the main effect of stimulus category.

11 In fact, we could not find any information about the strength of those associations, since existing norms in Hebrew are very limited with respect to the number of words they include.

12 Subsequent analysis of the participants’ answers to both questions revealed quite high values in the sensitivity measure (*A’* = 0.97 and 0.87 for questions 1 and 2, respectively), assuring that participants actually looked at and remembered the pictures as well as their color status (colored or black and white).

13 Similar to Experiment 3, the order of blocks in the blocked condition was counterbalanced between-participants by using a Latin square technique. A possible effect of order of blocks was tested by running a separate (order of blocks^∗^association strengths^∗^stimulus category) analysis of variance on the data from the blocked condition. The three-way and both two-way interactions elicited *F* < 1. The main effect of order of blocks was also not significant, *F*(1,34) = 1.04, *MSE* = 57,513, *p* = 0.42.

14 One of the reviewers suggested that a stronger magnitude of the lexical component observed in Experiments 3 and 4 compared to Experiments 1 and 2 may be due to a different type of letter string stimulus being used in these experiments. Thus, repeated-letter strings (e.g., rrrr) employed in Experiments 1 and 2 may be able to engage the articulation process more strongly than the various letter strings (e.g., zchma) used in Experiments 3 and 4. Although that might be true for other languages, it does not seem to fit what can be expected for the Hebrew language. According to Frost (2006), Hebrew is characterized by a deep orthography, meaning that some lexical information (i.e., vowels) is not present in written words and should be completed by the brain during the reading process. It is more probable, however, that the brain would attempt to complete the vowel information when faced with the various letter strings than with the repeated-letter strings because the former has a pattern more closely resembling real Hebrew words. The higher readability of the various letter strings, however, should be expressed in the inflated orthographic component and reduced lexical component. None of this, however, is consistent with the present results.
